# An encoding framework for binarized images using hyperdimensional computing

**DOI:** 10.3389/fdata.2024.1371518

**Published:** 2024-06-14

**Authors:** Laura Smets, Werner Van Leekwijck, Ing Jyh Tsang, Steven Latré

**Affiliations:** IDLab, Department of Computer Science, University of Antwerp-imec, Antwerp, Belgium

**Keywords:** hyperdimensional computing, vector symbolic architectures, image encoding, image classification, handwritten digit recognition

## Abstract

**Introduction:**

Hyperdimensional Computing (HDC) is a brain-inspired and lightweight machine learning method. It has received significant attention in the literature as a candidate to be applied in the wearable Internet of Things, near-sensor artificial intelligence applications, and on-device processing. HDC is computationally less complex than traditional deep learning algorithms and typically achieves moderate to good classification performance. A key aspect that determines the performance of HDC is encoding the input data to the hyperdimensional (HD) space.

**Methods:**

This article proposes a novel lightweight approach relying only on native HD arithmetic vector operations to encode binarized images that preserves the similarity of patterns at nearby locations by using point of interest selection and *local linear mapping*.

**Results:**

The method reaches an accuracy of 97.92% on the test set for the MNIST data set and 84.62% for the Fashion-MNIST data set.

**Discussion:**

These results outperform other studies using native HDC with different encoding approaches and are on par with more complex hybrid HDC models and lightweight binarized neural networks. The proposed encoding approach also demonstrates higher robustness to noise and blur compared to the baseline encoding.

## 1 Introduction

Because of the rising interest in the wearable Internet of Things (IoT), near-sensor artificial intelligence (AI) applications, and on-device processing, there is a considerable need for energy-efficient algorithms. Hyperdimensional computing (HDC), and in particular binary HDC, has been proposed in the literature as a brain-inspired, lightweight, and energy-efficient method because it has the advantages of few data requirements (Rahimi et al., 2019), robustness to noise (Kanerva, 2009; Widdows and Cohen, 2015; Rahimi et al., 2019), low latency (Rahimi et al., 2019), and fast processing (Rahimi et al., 2019). HDC maps input data to a hyperdimensional (HD) space in which information is distributed across thousands of vector elements, inspired by the large number of neurons that store information in the human brain. Since HDC uses simple HD arithmetic operations, it is computationally less complex than traditional deep learning (DL). HDC has already been used in several applications, such as speech recognition (Imani et al., 2017), human activity recognition (Kim et al., 2018), hand gesture recognition (Rahimi et al., 2016a; Moin et al., 2021; Zhou et al., 2021), text classification (Rachkovskij, 2007), classification of medical images (Kleyko et al., 2017a; Watkinson et al., 2021), character recognition (Manabat et al., 2019), robotics (Neubert et al., 2019), and time series classification (Schlegel et al., 2022).

A key aspect that determines the performance of HDC is encoding the input data to the HD space, which highly depends on the type of input data. To date, studies have clearly defined how text data (Rahimi et al., 2016b), numeric data (Imani et al., 2017; Kim et al., 2018), and time-series data (Rahimi et al., 2016a) can be encoded in a simple way using the HD arithmetic operations. However, what is still missing in the literature is a uniform framework to encode (binarized) images. Therefore, this article aims to propose a novel lightweight HD approach to encode binarized images relying only on native HD arithmetic vector operations. In this aspect, the current article brings forward the following novelties:

1. *Local linear mapping* is introduced as a novel mapping method for numeric data, whereby nearby numerical values are represented by similar HD vectors, and all other values by orthogonal HD vectors. In particular, we demonstrate its application for encoding positions in 2D images;
2. A parameterized framework to encode binary images into HD vectors is defined which uses point of interest (POI) selection as a local feature extraction method and unifies existing approaches for native HD encoding of images;
3. The proposed framework is applied on benchmark data sets, reaching 97.92% classification accuracy on MNIST and 84.62% accuracy on Fashion-MNIST.

This article is organized as follows: It begins with a brief description of the HDC model for classification. Afterward, *local linear mapping* for numeric data is defined and its application to 2D position encoding is illustrated. This is then followed by an overview of encoding approaches for binarized images found in the literature, the introduction of our parameterized unified framework, and a description of the performed experiments to test the proposed encoding framework. Section 3 presents the results which are discussed in the fourth section. Finally, the last section will concern the conclusions of the article.

## 2 Materials and methods

### 2.1 Hyperdimensional computing

HDC is a mathematical framework using HD vectors [i.e., vectors with very high dimension typically up to ten thousand, also called hypervectors (HVs)] and simple HD arithmetic vector operations to represent data. The focus of this article is on dense binary HVs (i.e., the elements are 0 or 1 with an equal probability of occurrence of both values) of dimension 10,000 (Kanerva, 2009; Kleyko et al., 2018). The analysis of data relies on the similarity between HVs which is calculated using the normalized Hamming distance between two binary HVs **v**_1_ and **v**_2_^1^:


(1) $$\begin{array}{l} s\left({\text{v}}_{1},{\text{v}}_{2}\right)=1-\frac{h\left({\text{v}}_{1},{\text{v}}_{2}\right)}{D} \end{array}$$

with *s* the similarity between **v**_1_ and **v**_2_, *D* the HV dimension and *h* the Hamming distance between **v**_1_ and **v**_2_:


(2) $$\begin{array}{l} h\left({\text{v}}_{1},{\text{v}}_{2}\right)=\overset{D}{\underset{d=1}{\sum }}\left({\text{v}}_{1}\left[d\right]\text{ XOR }{\text{v}}_{2}\left[d\right]\right). \end{array}$$

The HD arithmetic vector operations include: **(a) bundling** ⊕: B×H→B: (**B**, **v**) → **B**+**v** where $B={\mathbb{N}}^{D}$ and $H={\left\{0,1\right\}}^{D}$ (i.e., element-wise addition) after which the bundle **B** can be binarized into the HV **v** with the majority rule [.]:B→H: **B**→**v** according to:


(3) $$v[d]=[B[d]]=\{\begin{array}{ll} 1 & \text{if}B[d]>\frac{n}{2}\\ 0 & \text{if}B[d]<\frac{n}{2}\\ rand(0,1) & \text{if}B[d]=\frac{n}{2} \end{array}$$

with *n* the number of HVs bundled in **B** and *rand*(0, 1) means that the component **v**[*d*] is randomly assigned to 0 or 1 in the presence of ties; **(b) binding** ⊗: H×H→H: (**v**_1_, **v**_2_) → **v**_1_XOR**v**_2_; and **(c) permutation** ρ: H→H (i.e., cyclic shift in binary HDC).

**Figure 1:**
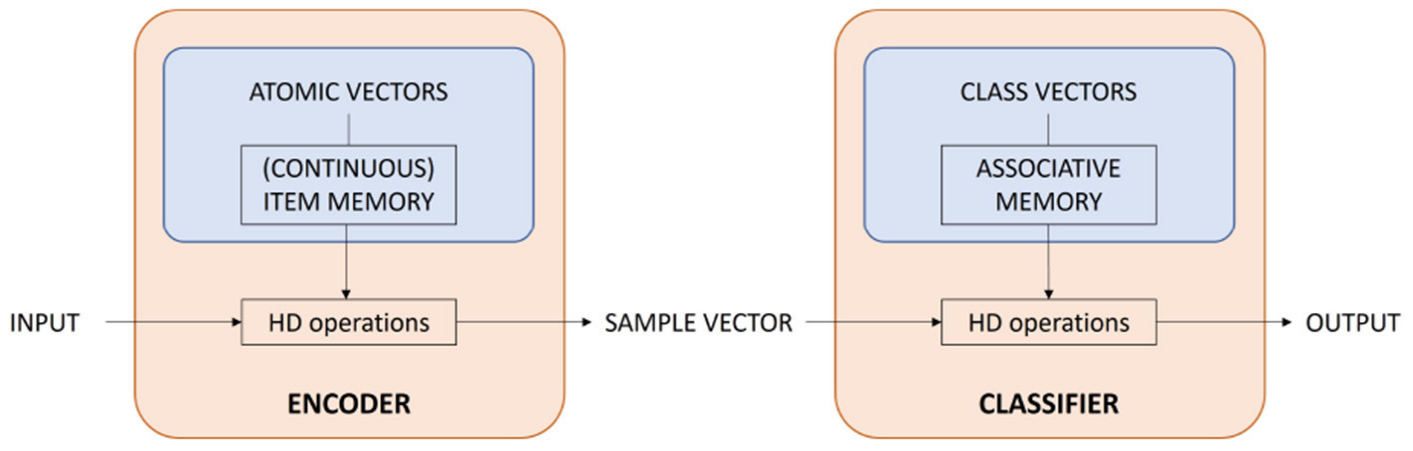
Schematic overview of the HDC framework in which two main building blocks can be distinguished: an encoder and a classifier.

Figure 1 gives a schematic overview of the framework of HDC in which two main building blocks can be distinguished: an encoder and a classifier. The encoder is responsible for mapping the input to an HV. Typically, it maps each input value of a sample to an atomic HV that is stored in (continuous) item memories ((C)IM). This procedure is called mapping and will be explained in Section 2.2. Then, different atomic HVs are combined using the HD vector operations to obtain one sample HV for each input.

**Figure 2:**
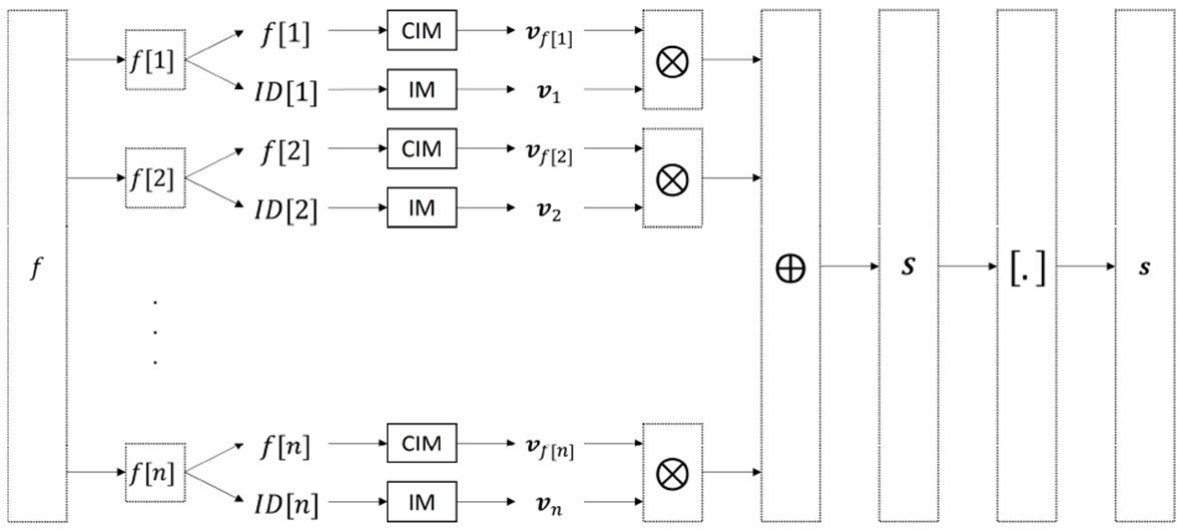
Schematic overview of the record-based encoding (Rachkovskij, 1990; Kussul and Rachkovskij, 1991; Imani et al., 2018) with an IM for the feature IDs and a CIM for the feature values.

Commonly, an input sample *f* having *n* features is encoded with the so-called record-based encoding (Rachkovskij, 1990; Kussul and Rachkovskij, 1991; Imani et al., 2018) as Figure 2: Each feature (*j* = 1...*n*) is assigned a random HV to represent the feature ID which is stored in an IM. Feature values are translated in HVs with a CIM that is created with *linear mapping* (see Section 2.2.2) (Rahimi et al., 2016a; Kleyko et al., 2018). Next, each feature ID HV **v**_*j*_ is bound with the HV representing its value **v**_*f*[*j*]_. Finally, these ID-value bound pairs of all features are bundled together to form the sample bundle **S** by initializing


(4) $$\begin{array}{l} {\text{B}}_{0}={\left\{0\right\}}^{D} \end{array}$$

and bundling each bound pair **v**_*f*[*j*]_⊗**v**_*j*_ one at a time:


(5) $$\begin{array}{l} {\text{B}}_{j}={\text{B}}_{j-1}⊕\left({\text{v}}_{f\left[j\right]}⊗{\text{v}}_{j}\right). \end{array}$$

The sample bundle **S** is then simply:


(6) $$\begin{array}{l} \text{S}={\text{B}}_{n}. \end{array}$$

For notation purposes, this iterative bundling (Equations 4–6) will be written in short as:


(7) $$\begin{array}{l} \text{S}=\overset{n}{\underset{j=1}{⊕}}\left({\text{v}}_{f\left[j\right]}⊗{\text{v}}_{j}\right) \end{array}$$

Finally, the sample bundle (Equation 7) is binarized into the HV **s** = [**S**] with the majority rule (Equation 3).

As the second main building block, the classifier has two modes of operation: (1) during training, the sample HVs and associated class labels are used to produce class prototypes by first bundling all sample HVs belonging to the same class and then updating these class bundles using misclassified samples; and (2) during inference, a sample HV is compared with each of the class prototypes and predicts the corresponding class label by selecting the class with highest similarity (Equations 1, 2). Different variants of training methods exist for which the interested reader is referred to our previous work (Smets et al., 2023) or the Supplementary material.

Since the encoder is a crucial part of the system, and a uniform framework to encode (binarized) images is still lacking in the literature, we propose a novel encoding framework (Section 2.3.2).

### 2.2 Data mapping techniques

#### 2.2.1 Orthogonal mapping

Orthogonal mapping assigns a randomly chosen atomic HV to each possible value present in the data. These random HVs are pseudo-orthogonal due to the high dimensionality which converges to exact orthogonality with increasing dimensionality (Kleyko et al., 2022). This type of mapping is suitable for nominal data where each value is independent from other values.

#### 2.2.2 *Linear mapping*

In the case of ordinal or discrete data, there is a natural ordering of levels or values such that closer levels should be mapped to more similar HVs than levels further apart, and similarity-preserving HVs are preferred for this type of data. Therefore, *linear mapping* of levels to atomic HVs is applied (Rahimi et al., 2016a; Kleyko et al., 2018). Namely, the lowest level is assigned a random atomic HV, whereafter each level's atomic HV is obtained by flipping $\frac{D/2}{L-1}$ bits in the atomic HV of the previous level, where L is the number of levels (without flipping a bit that has already been flipped before). Similarly, continuous data can be mapped to HVs with *linear mapping* after being quantized into a predefined number of discrete levels.

**Figure 3:**
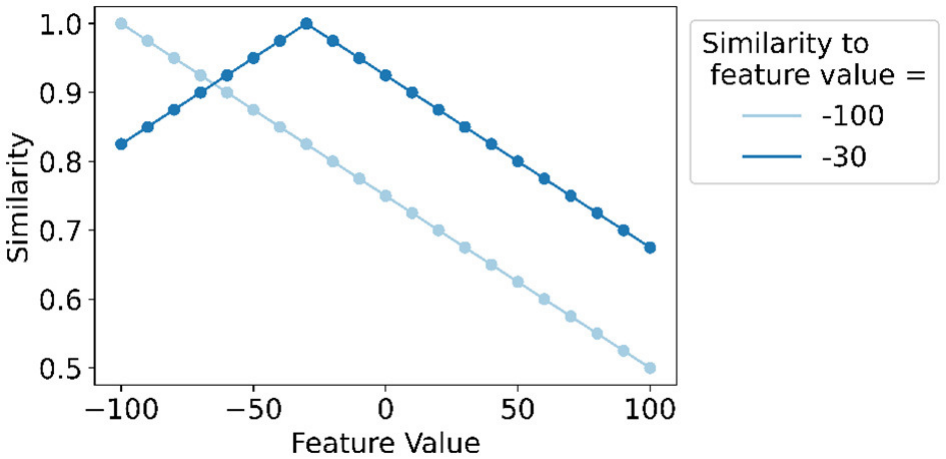
Example of *linear mapping* (Rahimi et al., 2016a; Kleyko et al., 2018) for a feature with discrete values ranging from −100 to 100 with steps of 10 and thus 21 levels. The similarity of each feature value's level hypervector to the lowest level hypervector (feature value = −100) and the hypervector for the feature value of −30 is shown.

As an example, Figure 3 illustrates the application of *linear mapping* for a feature with discrete values ranging from −100 to 100 with steps of 10 and thus 21 levels. It shows the similarity of values to the lowest level (feature value = −100) that decreases linearly up until orthogonality (similarity = 0.5) and the similarity of values to the feature value equal to −30 that decreases linearly for smaller and larger feature values.

#### 2.2.3 *Local linear mapping*

Encoding numeric data with original *linear mapping* results in small differences between the HVs of two adjacent values when working with a relatively large number of levels, and even values that are far apart are always somewhat similar (*s*>0.5). Therefore, we introduce *local linear mapping* which splits the range of values in *S* splits such that a smaller number of HVs (i.e., $\frac{L-1}{S}+1$ HVs) is present in each split to which *linear mapping* can be applied. As such, there are *S*+1 edge vectors, i.e., ${\text{v}}_{0},{\text{v}}_{\frac{L-1}{S}},{\text{v}}_{\frac{2\left(L-1\right)}{S}},...{\text{v}}_{L-1}$. The HV of the lowest level **v**_0_ is assigned randomly after which *linear mapping* is applied to the split between **v**_0_ and ${\text{v}}_{\frac{L-1}{S}}$ (Section 2.2.2) such that these two edge vectors are *D*/2 bits apart. The latter is then used to apply *linear mapping* in the following split, and so on. Consequently, two adjacent values within one split will have a larger difference in HVs [i.e., $\frac{D/2}{\left(\left(L-1\right)/S\right)}$ different bits] compared to when applying original *linear mapping* to the whole range of values. Additionally, an HV will be similar to HVs within a certain range from the considered HV and dissimilar, thus approximately orthogonal, to all HVs further away from the considered HV (i.e., outside that certain range). As a result, small differences in values are emphasized and large differences are ignored. Note that *local linear mapping* with 1 split or *L* splits correspond to the original *linear mapping* (Section 2.2.2) and orthogonal mapping (Section 2.2.1), respectively.

**Figure 4:**
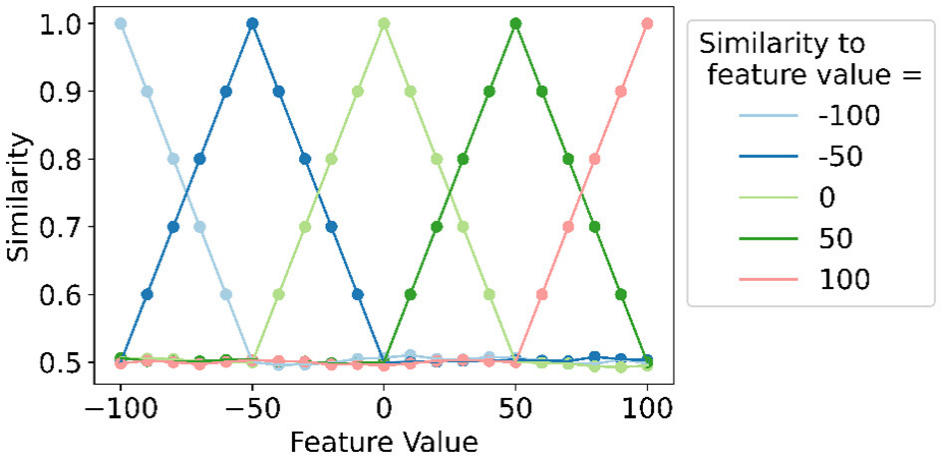
Example of *local linear mapping* with four splits for a feature with discrete values ranging from −100 to 100 with steps of 10 and thus 21 levels and $\frac{21-1}{4}+1=6$ levels in one split. The similarity of each feature value's level hypervector to each edge hypervector (feature value = −100, −50, 0, 50, and 100) is shown.

Figure 4 illustrates the concept of the proposed *local linear mapping* with four splits and thus six vectors in one split since there are 21 levels (i.e., $\frac{21-1}{4}+1$). In each of the four splits (e.g., between the edge vectors for values −100 and −50), original *linear mapping* is applied. Two adjacent values within one split will be highly similar; an HV will be similar to vectors at nearby positions to the left and right; an HV is orthogonal to vectors further to the left and right.

**Figure 5:**
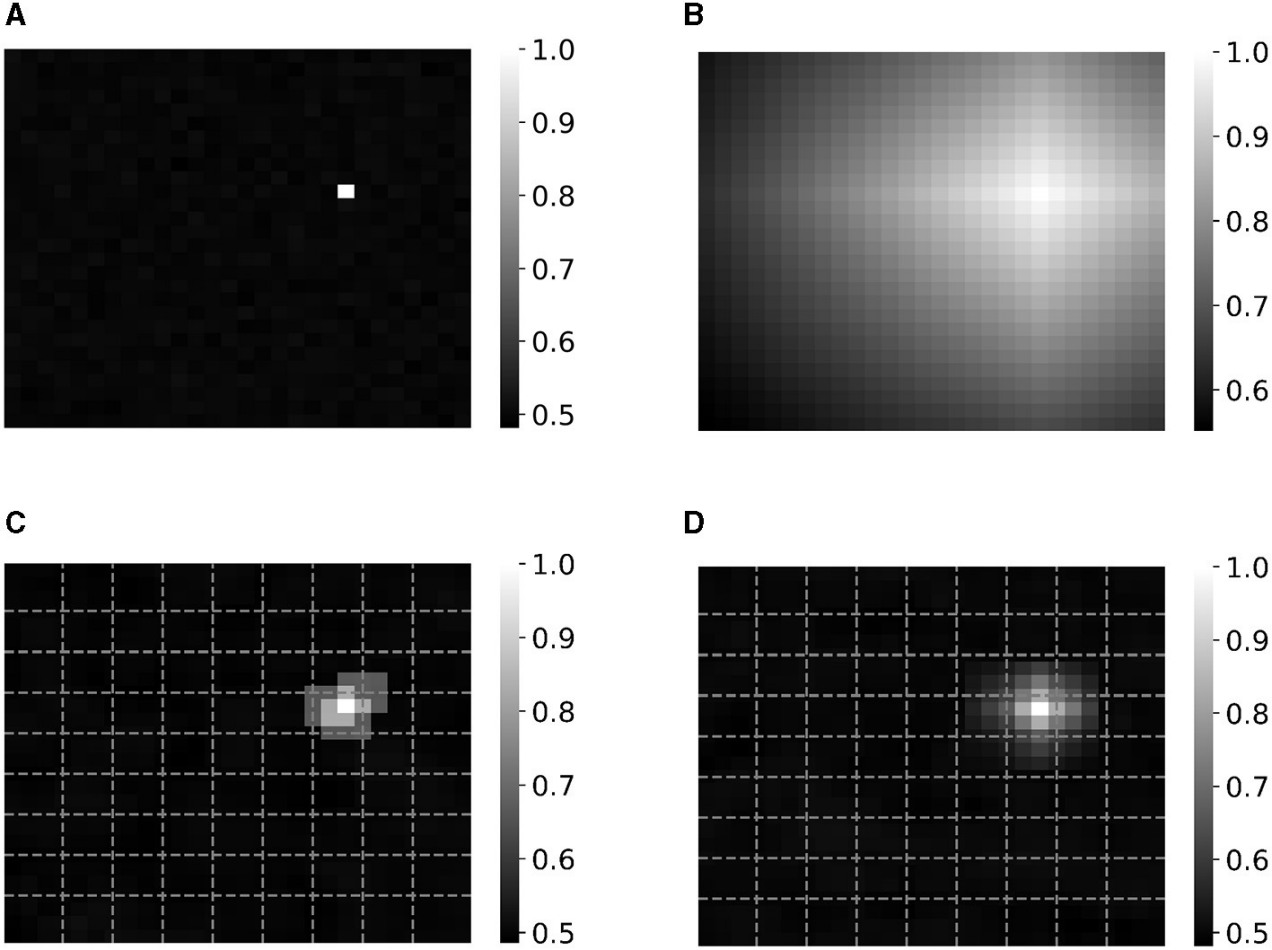
Similarity between all pixel's position vector and the position vector of the pixel at location (21,11) for an image of size 28-by-28 that are encoded as **v**_*x*_⊗**v**_*y*_ of which the *x* and *y* positions are mapped to vectors with **(A)** orthogonal mapping, **(B)** *linear mapping* (Rahimi et al., 2016a; Kleyko et al., 2018), **(C)** the concatenation approach of Rachkovskij et al. (2005) and Neubert and Schubert (2021) using 10 edge vectors for each axis (dotted lines) and **(D)** our proposed approach of *local linear mapping* with nine splits and thus 10 edge vectors (dotted lines).

*Local linear mapping* has some resemblance to a technique introduced by Rachkovskij et al. (2005) and Neubert and Schubert (2021) for encoding position in images, which concatenates orthogonal edge vectors *B*_*left*_ and *B*_*right*_ to obtain the position vectors within one split as [*B*_*left*_[1:α], *B*_*right*_[α+1:*end*]]. The ratio of concatenation α depends on the distance δ of the considered pixel to both edge vectors, and is equal to $D\cdot \frac{\delta_{right}}{\delta_{left}+\delta_{right}}$. However, the decrease in similarity for pixels further away from the considered pixels is not as gradual as with the proposed *local linear mapping*. This is shown in Figure 5 which illustrates the difference in similarity between all pixels' position HV and the position HV of the pixel at location (21,11) for an image of size 28-by-28. The position HVs are all encoded as **v**_*x*_⊗**v**_*y*_ of which the x and y positions are mapped to vectors **v**_*x*_ and **v**_*y*_ using the different types of mapping: (1) orthogonal mapping, (2) *linear mapping* (Rahimi et al., 2016a; Kleyko et al., 2018), (3) the concatenation approach of Rachkovskij et al. (2005) and Neubert and Schubert (2021) using 10 edge vectors and (4) our proposed *local linear mapping* using nine splits and thus also 10 edge vectors. We believe that the decrease in similarity for *local linear mapping* in Figure 5D is more intuitive than for the concatenation approach (Rachkovskij et al., 2005; Neubert and Schubert, 2021) (Figure 5C). Furthermore, *local linear mapping* builds further on the concept of *linear mapping* which is commonly used in HDC encoding approaches. In this aspect, it is also similar to float code, which builds further on thermometer code by making the similarity decay local (Rachkovskij et al., 2005; Frady et al., 2021).

### 2.3 Encoding techniques for binary images

#### 2.3.1 Related work

Several ways to encode binarized images with HDC have been proposed in the literature and can be divided into two main categories: (1) native HDC, i.e., end-to-end use of native HD vector operations (from raw pixel to output), and (2) hybrid HDC, i.e., external feature extraction methods are used in combination with HDC. Table 1 gives an overview of the different encoding approaches which are discussed in the following section.

**Table 1:** Summary of the already proposed approaches for the encoding of binarized images.

**Description**				**Encoded image v_*I*_**
Native	Ortho-gonal	Permutation	1D^a^	$\left[{⊕}_{x=1}^{w*h}\left(\rho^{p\left[x\right]}{\text{v}}_{x}\right)\right]$
			2D^b^	$\left[{⊕}_{x=1}^{w}{⊕}_{y=1}^{h}\left(\rho_{X}^{x}\rho_{Y}^{y}{\text{v}}_{I_{bin}\left[x,y\right]}\right)\right]$
		Binding	1D^c^	$\left[{⊕}_{x=1}^{w*h}\left({\text{v}}_{x}⊗{\text{v}}_{p\left[x\right]}\right)\right]$
			2D^d^	$\left[{⊕}_{x=1}^{w}{⊕}_{y=1}^{h}\left({\text{v}}_{x}⊗{\text{v}}_{y}⊗{\text{v}}_{I_{bin}\left[x,y\right]}\right)\right]$
		Both	1D^e^	$\left[{⊕}_{x=1}^{\left(w*h\right)-n+1}\left({\text{v}}_{x}⊗{⊗}_{j=0}^{n-1}\left(\rho^{j}{\text{v}}_{x\left[i+j\right]}\right)\right)\right]$
	Linear	Binding	2D^f^	$\left[{⊕}_{x=1}^{w}{⊕}_{y=1}^{h}\left({\text{v}}_{x}⊗{\text{v}}_{y}⊗{\text{v}}_{I_{bin}\left[x,y\right]}\right)\right]$
Hybrid	Feature extraction's output^g^			**v**_*output*_
	Record-based feature encoding^h^			$\left[{⊕}_{i=1}^{n}\left({\text{v}}_{f\left[i\right]}⊗{\text{v}}_{i}\right)\right]$

The table includes a short description of the type of encoding, and the formula to obtain the encoded HV of the image. The symbols used in this table are listed in Supplementary Table S1.

^a^Kleyko et al. (2016, 2017b), Manabat et al. (2019), and Hassan et al. (2022).

^b^Kussul et al. (2006), Mitrokhin et al. (2019), Kleyko et al. (2020), and Rachkovskij (2022).

^c^Yang and Ren (2017), Ma et al. (2021), Watkinson et al. (2021), Bosch et al. (2022), Duan et al. (2022a), and Ma and Jiao (2022).

^d^Kelly et al. (2013).

^e^Khaleghi et al. (2022).

^f^Kussul et al. (1992), Gallant and Culliton (2016), and Weiss et al. (2016).

^g^Yilmaz (2015), Kleyko et al. (2017a), Karvonen et al. (2019), and Zou et al. (2021a).

^h^Kussul and Rachkovskij (1991).

##### 2.3.1.1 Native HDC

Assume an image *I* of size *w*×*h* is given as an input which is binarized, denoted here as *I*_*bin*_. The binarized image is either flattened into an array *p* of length w*h where *p*[*x*] is the value of the pixel in the array *p* at position *x* or used in its original 2D format where *I*_*bin*_[*x, y*] is the value of the pixel in the binary image *I*_*bin*_ at position (*x, y*).

The native HDC encoding methods can be further divided into two categories depending on whether the position is encoded while preserving similarity between nearby positions (i.e., linearly mapped) or not (i.e., orthogonally mapped).

###### 2.3.1.1.1 Orthogonally mapped position vectors

*(a) Permutation*. When considering the flattened image, a unique random HV is assigned to each pixel position in the array *p* after which the obtained position HV **v**_*x*_ is shifted with one position if the corresponding pixel value *p*[*x*] is one and not shifted if it is zero (Kleyko et al., 2016, 2017b; Manabat et al., 2019; Hassan et al., 2022). To encode the 2D binarized image, two unique permutations ρ_*X*_ and ρ_*Y*_ are assigned to represent the *x*- and *y*-axis of the image, respectively. These permutations are applied *x* and *y* times, respectively, to the pixel value HV **v**_*I*_*bin*_[*x, y*]_ (Kussul et al., 2006; Mitrokhin et al., 2019; Kleyko et al., 2020; Rachkovskij, 2022).

*(b) Binding*. A unique random HV is assigned to each possible pixel value (i.e., zero and one). Thereafter, the pixel value HV **v**_*p*[*x*]_ or **v**_*I*_*bin*_[*x, y*]_ is bound with its corresponding position HV **v**_*x*_ or (**v**_*x*_⊗**v**_*y*_) for the flattened image (Yang and Ren, 2017; Ma et al., 2021; Watkinson et al., 2021; Bosch et al., 2022; Duan et al., 2022a; Ma and Jiao, 2022) or 2D image (Kelly et al., 2013), respectively, which are mapped orthogonally.

*(c) Combination of permutation and binding*. In analogy to the *n*-gram encoding in language identification applications (Rahimi et al., 2016b), Khaleghi et al. (2022) apply a sliding window of length *n* to the image. The window is then encoded by binding all pixel value HVs which are permuted based on the position in the window, i.e., the first pixel value's HV is not permuted, the second is permuted once, the third is twice permuted, etc. This could be seen as extracting local features from the image. To account for the global position of these features in the image, each window HV is bound with a random position HV.

The encoding approaches mentioned so far represent similar pixels at nearby positions by dissimilar HVs, because of the property of permutation that a permuted HV is dissimilar to its original, and because of orthogonal position HVs. Hence, these encoding approaches do not preserve similarity which might be crucial to solving an image classification task.

###### 2.3.1.1.2 Linearly mapped position vectors

Kussul et al. (1992), Gallant and Culliton (2016), and Weiss et al. (2016) apply *linear mapping* such that nearby *x* and *y* positions are represented by similar HVs. The image is then encoded using the binding operation for a 2D image, as mentioned in Section 2.3.1.1.1(b).

An alternative approach to preserving similarity for nearby positions is proposed by Komer et al. (2019), Voelker et al. (2021), and Frady et al. (2022) who make use of fractional binding. For this, two random HVs **x** and **y** are assigned to represent the x- and y-axis, respectively. The (*x, y*) position is then constructed as **x**^*x*^⊗**y**^*y*^ where ${\text{x}}^{x}={⊗}_{n=1}^{x}\text{x}$, i.e., the HV **x** is repeatedly bound with itself *x* times. This bound pair representing the position is then bound with the pixel value HV **v**_*I*_*bin*_[*x, y*]_. However, this type of position encoding cannot be applied to binary HVs since binding in binary HDC is performed with XOR such that a binary HV bound with itself for an even or odd amount of times results in an HV containing all zeros or the original binary HV, respectively.

##### 2.3.1.2 Hybrid HDC

Instead of encoding the raw image using HD vector operations, external non-HD-based feature extraction methods are used. These approaches can be subdivided into two categories: **(a)** those that use the output layer of a neural network (NN) or cellular automata (CA) as single feature HV to represent the image (Yilmaz, 2015; Kleyko et al., 2017a; Karvonen et al., 2019; Zou et al., 2021a); and **(b)** those that use external methods (NN or other) to extract multiple features which are encoded via the record-based encoding (Figure 2) (Kussul and Rachkovskij, 1991).

#### 2.3.2 Proposed unified framework

**Figure 6:**
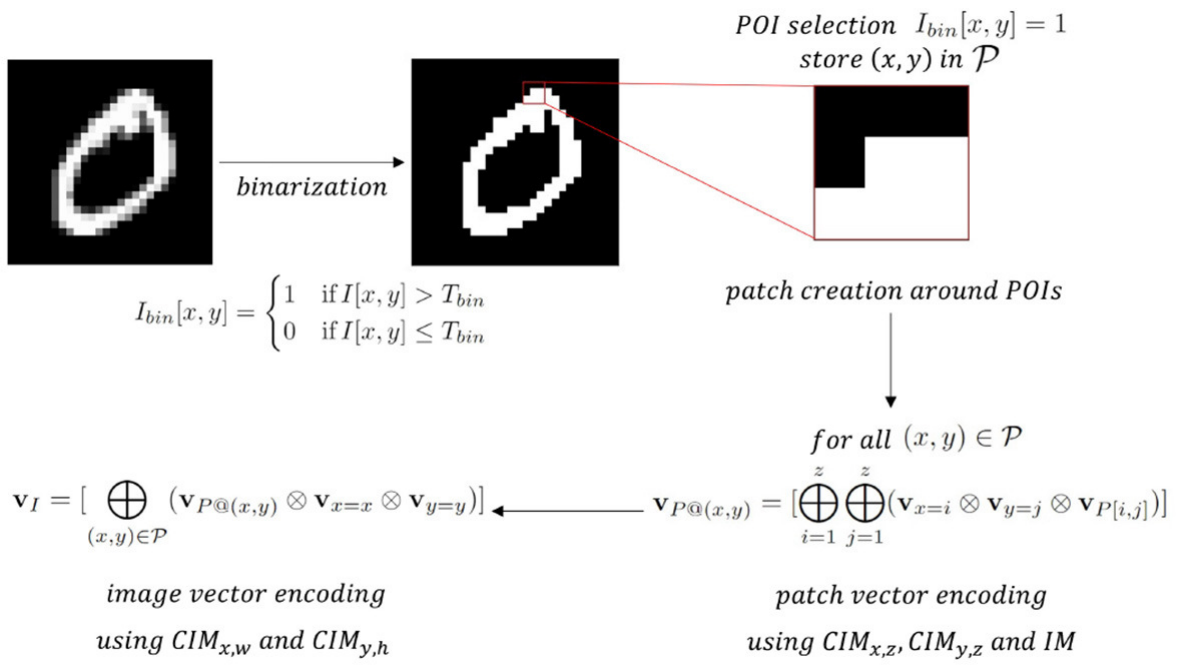
Schematic overview of the proposed unified encoding framework for a training sample of the MNIST data set with size 28-by-28 using a patch size of 3-by-3 around the POIs (*z* = 3, *h* = 28 and *w* = 28).

Figure 6 gives an overview of the proposed approach to encode binarized images which can be divided into four steps: (1) binarization, (2) POI selection and patch creation around POIs, (3) patch vector encoding, and (4) image vector encoding.

##### 2.3.2.1 Binarization

As a first step, the pixel values of an input image *I* are binarized using a predefined binarization threshold *T*_*bin*_:


(8) $$I_{bin}[x,y]=\{\begin{array}{ll} 1 & \text{if}I[x,y]>T_{bin}\\ 0 & \text{if}I[x,y]\leq T_{bin} \end{array}$$

##### 2.3.2.2 POI selection and patch creation around POIs

Points of interest (POIs) are selected as pixels with *I*_*bin*_[*x, y*] = 1. Thereafter, a square patch *P* of predefined size *z* is drawn around each POI (in Figure 6, *z* = 3).

##### 2.3.2.3 Patch vector encoding

Each pixel in the patch is encoded as the binding of three vectors: the HV representing its binary value *P*[*x, y*] (stored in *IM*, one random vector for value 0 and another random vector for value 1), the HV corresponding to its *x* position in the patch and the one for the y position in the patch. The *x* and *y* position HVs are stored in two separate CIMs (*CIM*_*x, z*_ and *CIM*_*y, z*_), both containing *z* vectors that are mapped with orthogonal mapping. The resulting patch vector for the POI with position (x,y) is then obtained by bundling all pixel vectors and binarizing the obtained bundle with majority rule (Equation 3):


(9) $$\begin{array}{l} {\text{v}}_{P@\left(x,y\right)}=\left[\overset{z}{\underset{i=1}{⊕}}\overset{z}{\underset{j=1}{⊕}}\left({\text{v}}_{x=i}⊗{\text{v}}_{y=j}⊗{\text{v}}_{P\left[i,j\right]}\right)\right] \end{array}$$

for all (x,y)∈P. The encoding of patch vectors around POIs can be seen as extracting local features of the image in analogy to Kussul and Baidyk (2004), Kussul et al. (2006), and Curtidor et al. (2021), but here only native HD arithmetic operations are used instead of relying on an NN-based feature extractor.

##### 2.3.2.4 Image vector encoding

After obtaining the patch vectors of all POIs with Equation 9, each patch vector is bound with the HVs representing the corresponding POI's x and y position in the original image *I* (stored in *CIM*_*x, w*_ and *CIM*_*y, h*_) to capture the global positional information of the extracted local features. The binarized bundling of all these patch vectors bound with its POI's position results in the image vector:


(10) $$\begin{array}{l} {\text{v}}_{I}=\left[\underset{\left(x,y\right)\in P}{⊕}\left({\text{v}}_{P@\left(x,y\right)}⊗{\text{v}}_{x=x}⊗{\text{v}}_{y=y}\right)\right] \end{array}$$

The *CIM*_*x, w*_ and *CIM*_*y, h*_ are mapped with our proposed *local linear mapping* (Section 2.2.3) instead of original *linear mapping* to capture small dependencies in position while ignoring large ones.

### 2.4 Experiments

The abovementioned proposed approach to encode binarized images is tested on two known, publicly available data sets: (1) MNIST data set (LeCun et al., 1998) which includes 70,000 28-by-28 grayscale images of ten different handwritten digits; and (2) Fashion-MNIST data set (Xiao et al., 2017) containing 7,000 28-by-28 grayscale images of fashion products for each of ten categories, i.e., 70,000 images in total. Both data sets are split into a training set of 60,000 images (6,000 for each class) and a test set of 10,000 images (1,000 for each class). The pixel values range from 0 to 255.

#### 2.4.1 *Local linear mapping*

At first, the concept of *local linear mapping* is tested using pixel-wise encoding on the whole image, without using POI encoding. The image is thus encoded as:


(11) $$\begin{array}{l} {\text{v}}_{I}=\left[\overset{w}{\underset{x=1}{⊕}}\overset{h}{\underset{y=1}{⊕}}\left({\text{v}}_{x}⊗{\text{v}}_{y}⊗{\text{v}}_{I_{bin}\left[x,y\right]}\right)\right] \end{array}$$

The number of splits *S* in the CIMs storing **v**_*x*_ and **v**_*y*_ is treated as a hyperparameter and tested for the settings *S* = {1, 2, 3, 4, 5, 6, 7, 8, 9, 28} of which the second from last is the maximal number of splits possible for a 28-by-28 image, since otherwise only two vectors would be in a particular split and thus will be orthogonal. Note again that using only one split corresponds to the traditional *linear mapping* (Section 2.2.2) and will be treated as the baseline HDC framework, and using 28 splits corresponds to orthogonal mapping (Section 2.2.1). The images are binarized following Equation 8 with the binarization threshold equal to zero (i.e., *T*_*bin*_ = 0).

#### 2.4.2 Proposed unified framework

In the second part of the experiments, *local linear mapping* is applied in combination with the POI encoding (Equation 11). This encoding approach requires determining the settings of two hyperparameters: the number of splits for *local linear mapping* *S* and the patch size *z* around each POI. All possible combinations of the following settings of the two hyperparameters are tested: *S* = {1, 2, 3, 4, 5, 6, 7, 8, 9, 28} and *z* = {3, 5, 7}. The images are again binarized following Equation 8 with the binarization threshold equal to zero (i.e., *T*_*bin*_ = 0).

#### 2.4.3 Hyperparameter selection

The different combinations of settings are tested using 10-fold cross-validation (CV) on the training set. This means that the 60,000 training images are split into ten parts. The algorithm is trained on 54,000 images and validated on the remaining 6,000 images which is repeated ten times each time taking a different set of 6,000 validation images. The training procedure is performed iteratively for a maximum of 1,000 iterations while saving the classifier with the best accuracy. After every 100 iterations, we evaluate whether this best training accuracy exceeds 99% accuracy. If this is the case, the training procedure is terminated and the classifier with the best accuracy is used on the validation set. The performance of the HDC classifier for each combination of hyperparameter settings is reported as the average validation accuracy over the ten folds of the 10-fold CV.

#### 2.4.4 Evaluation on the test set

The combination of hyperparameter settings yielding the largest average validation accuracy is selected for both the MNIST and Fashion-MNIST data sets. These settings are used to train the classifier using the entire training set (i.e., all 60,000 images). Contrary to the CV experiments (Section 2.4.3), the training procedure for MNIST is only terminated when the best training accuracy exceeds 99.9% accuracy, and the maximal amount of iterations in the training procedure for Fashion-MNIST is increased to 2,000 iterations. Afterward, the trained classifier is tested on the 10,000 test images. This process is repeated for ten independent runs across which the average test accuracy is calculated.

#### 2.4.5 Robustness analysis

To test the robustness to noise and blur of the proposed encoding approach, the MNIST-C data set which is proposed as a robustness benchmark for computer vision by Mu and Gilmer (2019) is used. This data set includes the 60,000 training and 10,000 test images of the original MNIST data set (LeCun et al., 1998) to which several different corruptions are applied, including shot noise, impulse noise, glass blur, motion blur, and spatter which are of particular interest in the current article to test noise and blur robustness. The HDC model with the proposed encoding is trained on the original 60,000 training images (i.e., without corruptions) with the baseline setting of hyperparameters (*S* = 1 and no POI selection, Equation 10) and the setting yielding the best validation accuracy after 10-fold CV (Section 2.4.3, Equation 11). Both trained HDC classifiers are then tested on the five selected corrupted test sets of 10,000 images for which a test accuracy averaged over ten independent runs is calculated.

## 3 Results

### 3.1 *Local linear mapping*

**Figure 7:**
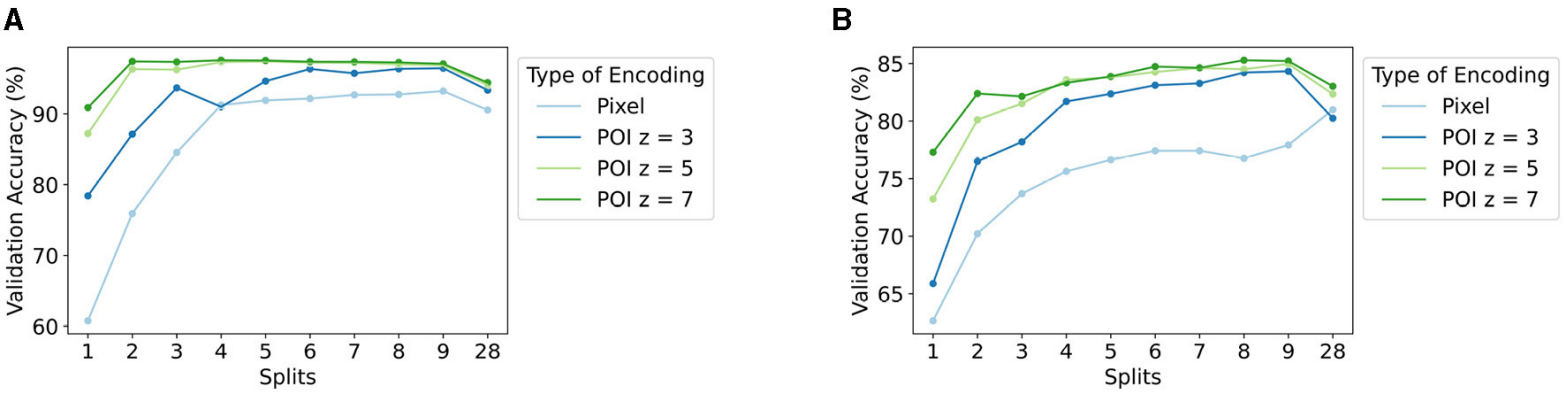
Accuracy (%) on the validation set averaged over the ten folds of 10-fold cross-validation for the **(A)** MNIST and **(B)** Fashion-MNIST data sets, for the different settings of the number of splits *S* used in *local linear mapping*, and for pixel-wise (Equation 10) and POI (Equation 11) encoding with different patch sizes *z*.

The results of the experiments testing the effect of the number of splits in *local linear mapping* using pixel-wise encoding (Equation 10) are presented in light blue in Figure 7 (see also Supplementary Table S2). The figure shows the accuracy on the validation set averaged over the ten folds of the 10-fold CV for both the MNIST and Fashion-MNIST data sets. As mentioned previously, the number of splits equal to 1 (*S* = 1) is treated as our baseline since this does not use *local linear mapping* nor POI encoding. As such, the baseline average validation accuracy is 60.78% for MNIST and 62.65% for Fashion-MNIST.

An increase in performance is seen when increasing the number of splits used in *local linear mapping* from 1 to 9. The largest validation accuracy is 93.21% for MNIST for *S* = 9 and 80.98% for Fashion-MNIST for *S* = 28, which is an increase of 32.43 and 18.33%, respectively. In the case of MNIST, the classifier with orthogonal mapping (*S* = 28) reaches an accuracy that is slightly lower than the largest obtained accuracy, while this setting yields the highest accuracy for Fashion-MNIST.

### 3.2 Proposed unified framework

Figure 7 also shows the results illustrating the effect of the two hyperparameters (i.e., the number of splits *S* in *local linear mapping* and the patch size *z* in POI encoding) for our proposed encoding approach (Equation 11, see also Supplementary Table S3). The figure again includes the accuracy on the validation set averaged over the ten folds of the 10-fold CV for both the MNIST and Fashion-MNIST data sets.

Similar to the previous section, there is a clear trend of increasing validation accuracy with an increasing number of splits *S* used in *local linear mapping* up until *S* = 9, followed by a small drop for *S* = 28. An increase in performance is also seen with increasing patch size *z*. Interestingly, the influence of the number of splits *S* on the performance seems to decrease for a larger patch size *z*.

The best achieved validation accuracy is 97.56% for MNIST with *S* = 4 and *z* = 7 and 85.28% for Fashion-MNIST with *S* = 8 and *z* = 7. This corresponds to an increase in performance of 36.78% for MNIST and 22.63% for Fashion-MNIST compared to their baseline accuracy (*S* = 1 and pixel-wise encoding in Figure 7). These settings for the two hyperparameters yielding the best validation accuracy are used to test the HDC classifier on the test set in the next section.

### 3.3 Evaluation on the test set

Table 2 shows the results obtained when setting the hyperparameters to the values yielding the best validation accuracy obtained in the previous section (Section 3.2). The table presents the accuracy on the entire training set, the accuracy on the unseen test set and the number of iterations needed to obtain the best training accuracy, averaged over ten independent runs. An average accuracy of 97.92% is reached on the test set of MNIST. For the Fashion-MNIST data set, an average test accuracy of 84.62% is obtained.

**Table 2:** Accuracy (%) on the full training and unseen test set and the number of iterations needed to reach the best training accuracy, averaged over ten independent runs for the MNIST (*S* = 4 and *z* = 7) and Fashion-MNIST (*S* = 8 and *z* = 7) data sets.

**MNIST**			**Fashion-MNIST**		
**Training accuracy**	**Test accuracy**	**Iteration**	**Training accuracy**	**Test accuracy**	**Iteration**
99.92 (± 0.02)	97.92 (± 0.07)	509 (± 90)	87.60 (± 0.01)	84.62 (± 0.01)	1606 (± 266)

Data are mean (±standard deviation).

### 3.4 Robustness analysis

**Figure 8:**
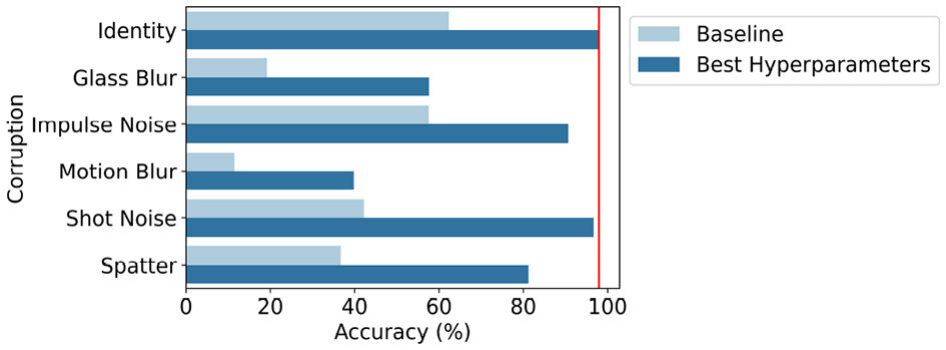
Accuracy (%) on the identity test set and five selected corrupted test sets of MNIST-C, averaged over ten independent runs with the hyperparameters set to the baseline setting (*S* = 1 and no POI selection) and the setting yielding the best validation accuracy with 10-fold CV (*S* = 4 and *z* = 7). In red is the accuracy on the identity test set with the optimal hyperparameters.

Figure 8 sets out the results obtained during the analysis of robustness to noise and blur. The figure shows the accuracy on the original (i.e., identity corruption and red line in the figure) and five selected corrupted test sets, averaged over ten independent runs for the MNIST-C data set with the hyperparameters set to the baseline setting (*S* = 1 and no POI selection, Equation 10) and the setting yielding the best validation accuracy with 10-fold CV (*S* = 4 and *z* = 7, Equation 11 and Section 3.2). More detailed results can be found in the Supplementary Table S4. To conclude, it can be seen that the best hyperparameters setting achieves an average test accuracy of 73.20% for the five corrupted test sets, which is an increase of 39.77% compared to the baseline setting which achieves 33.44% average test accuracy.

## 4 Discussion

### 4.1 Analysis of results

The results in Figure 7 for pixel-wise encoding show that the proposed *local linear mapping* for position encoding outperforms *linear mapping*. More specifically, there is an increase in performance with an increasing number of splits used in *local linear mapping*. This interesting finding indicates the importance of discriminating better between smaller differences in position in the image instead of larger differences. This is a result of the splits in *local linear mapping* that represent two positions that are far apart with orthogonal HVs, and only HVs of close positions are similar. By contrast, in *linear mapping*, the HVs of both close and far positions have a certain degree of similarity.

Another finding that stands out from the results is a remarkable increase in performance when encoding patches around POIs compared to pixel-wise encoding which becomes even more prominent with an increasing patch size (Figure 7). Several factors could explain this observation. Firstly, background pixels are ignored with POI encoding, limiting unnecessary information. Secondly, local features are extracted around each POI such that the local neighborhood of each POI is taken into account.

In addition, employing *local linear mapping* to encode the global position of POIs in the image improves the performance compared to using *linear mapping* (Figure 7). This finding is in line with the results obtained with pixel-wise encoding and can be explained similarly.

Finally, the results of the robustness analysis indicate that the proposed encoding approach after hyperparameter selection shows higher robustness to noise and blur than the baseline HDC encoding approach (Section 4.3 and Supplementary Table S4).

### 4.2 Comparison to the state-of-the-art

#### 4.2.1 MNIST data set

**Figure 9:**
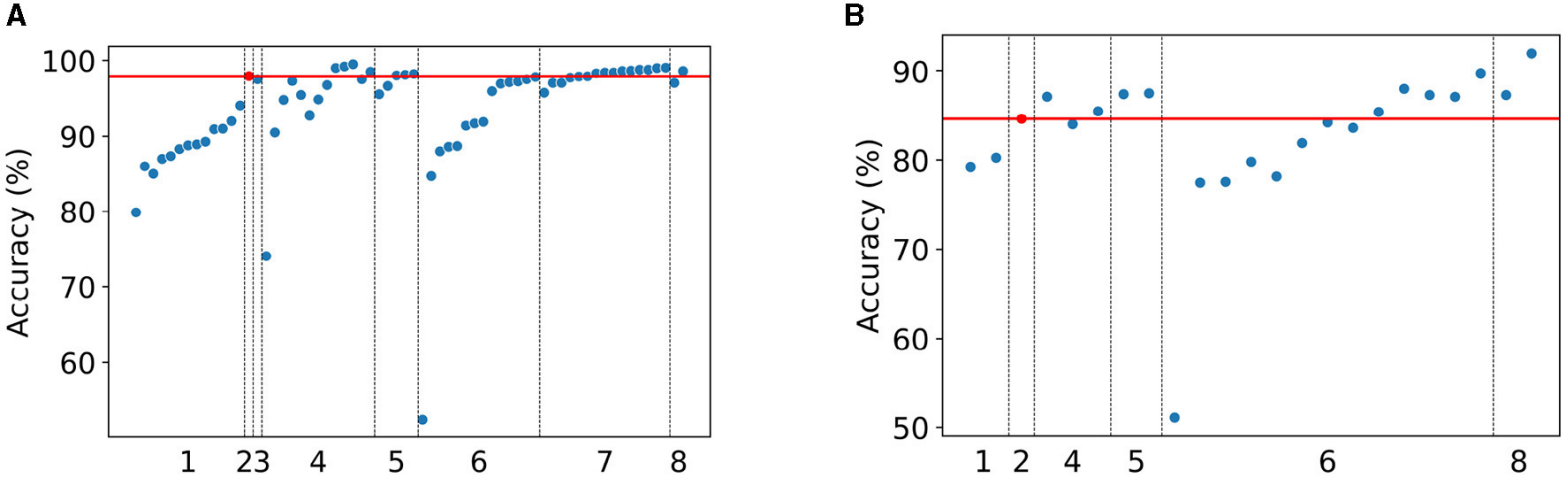
Comparison of our proposed framework to the results of studies found in the literature. **(A)** MNIST. **(B)** Fashion-MNIST. Our obtained accuracy of 97.92% for MNIST and 84.62% for Fashion-MNIST are highlighted with the red dot and red line. The results are categorized into the categories: (1) Native HDC, (2) Our proposed framework, (3) Adaptive HDC, (4) Hybrid HDC, (5) Multi-bit HDC, (6) Traditional Machine Learning, (7) Binary Neural Networks, and (8) Binary Spiking Neural Networks.

Figure 9A compares our obtained result for MNIST (i.e., 97.92%) to the results of other studies found in the literature (see also Supplementary Table S5).

The proposed approach of POI encoding with *local linear mapping* outperforms all methods categorized in *Native HDC*. This includes the methods applying the permutation operation to encode the position of pixels in the flattened image [Section 2.3.1.1.1(a)], i.e., Manabat et al. (2019) and Hassan et al. (2022) report an accuracy of 79.87 and 86%, respectively. Our obtained result for MNIST is also better compared to several studies using the binding operation for position encoding in the flattened image [Section 2.3.1.1.1(b)]. Namely, Chuang et al. (2020), Chang et al. (2021), Hernández-Cano et al. (2021), Hsieh et al. (2021), Kazemi et al. (2021), Zou et al. (2021b), Bosch et al. (2022), Duan et al. (2022a,b), and Ma and Jiao (2022) report baseline accuracies ranging from 85 to 92%. In addition, the *n*-gram-based encoding method to extract local features by Khaleghi et al. (2022) reaches an accuracy of 94.0% which we outperform by using *local linear mapping* instead of orthogonal mapping to encode global positional information.

Hernández-Cano et al. (2021) propose OnlineHD that can increase their baseline performance of 91 to 97%, which is lower than our obtained accuracy. In OnlineHD, the baseline HDC training procedure is extended by updating the HDC model depending on how similar a sample is to the existing model. As such, the training procedure becomes more complex due to floating-point multiplications. OnlineHD is categorized as *Adaptive HDC*.

Other studies use the HDC framework in combination with additional non-HD methods (*Hybrid HDC*, Section 2.3.1.2), such as elementary CA which is used to derive the high-dimensional vector by Karvonen et al. (2019) resulting in an accuracy of 74.06%. Zou et al. (2021a) extracts low-level features with an SNN before using HDC reaching an accuracy of 90.5%. Duan et al. (2022a) and Yan et al. (2023) employ binary neural networks (BNN) in combination with HDC reaching an accuracy of 94.74 and 97.25%, respectively. Random Fourier Features (RFF) are used by Yu et al. (2022) for the encoding of the images resulting in 95.4% accuracy. Traditional NNs have also been combined with HDC resulting in accuracies of 92.72% (Duan et al., 2022b), 94.8% (Liang et al., 2022), and 96.71% (Ma and Jiao, 2022). Zou et al. (2021b) report an accuracy of 97.5% by extending the HDC encoding framework with manifold learning. Our proposed encoding approach using only native HD vector operations outperforms these hybrid HDC methods. Nevertheless, other hybrid HDC methods obtain better results. Poduval et al. (2021) extract features from the original images and apply record-based encoding obtaining a performance of 99%. Kussul and Baidyk (2004) and Kussul et al. (2006) reach a higher accuracy of 99.2 and 99.5% with the NN-based local feature extraction. Rachkovskij (2022) extracts local binary pattern (LBP) features, proposes a shift-equivariant similarity-preserving scheme for position encoding, and uses a large margin perceptron for classification reaching an accuracy of 98.5% with a vector dimension of 10,000.

Several works increase the complexity of HDC by using multi-bit representations (i.e., *Multi-bit HDC*) instead of single-bit (i.e., binary). Imani et al. (2019), Chuang et al. (2020), Kazemi et al. (2021), Kim et al. (2021), and Yu et al. (2022) use vectors with more complex elements achieving 95.5, 96.6, 98, 98.09, and 98.2%, respectively. With only the latter three achieving slightly higher accuracy than ours, we can conclude that our proposed binary, native HDC method using *local linear mapping* and POI encoding achieves comparable results with these more complex multi-bit HDC methods.

Even though this article aims to improve native HDC encoding of binarized images, we compare the proposed encoding method to lightweight *Non-HDC* methods. Results for a wide range of traditional machine learning (ML) methods including decision tree, multi-layer perceptron, and support vector classification are reported by Xiao et al. (2017). The proposed HDC framework outperforms all these ML methods with accuracies ranging from 52.4 to 97.8%, including the AdaBoost classifier of Kim et al. (2017). Several studies employ BNNs to solve the MNIST classification task obtaining accuracies in the range of 95.7 and 99.04% (Cheng et al., 2015; Courbariaux et al., 2015, 2016; Kim and Smaragdis, 2015; McDanel et al., 2017; Umuroglu et al., 2017; Yang et al., 2017; Chi and Jiang, 2018; Ghasemzadeh et al., 2018; Jokic et al., 2018; Narodytska et al., 2018; Sun et al., 2018; Valavi et al., 2018; Simons and Lee, 2019; Yan et al., 2023). Finally, binary spiking neural networks (SNN) reach accuracies of 97.0–98.6% (Kheradpisheh et al., 2021; Mirsadeghi et al., 2023). To conclude, our obtained result of 97.92% for the MNIST data set outperforms native HDC methods and is on par with more complex hybrid HDC or lightweight non-HDC methods.

#### 4.2.2 Fashion-MNIST data set

Figure 9B compares our obtained result for Fashion-MNIST (i.e., 84.62%) to the results of other studies found in the literature (see also Supplementary Table S5).

There are not as many studies available for the Fashion-MNIST data set as for MNIST. Duan et al. (2022a,b) report an accuracy of 79.24 and 80.26% for *Native HDC*. Using *Hybrid HDC* methods, Yu et al. (2022) report an accuracy of 84.0% using RFF and reach 87.4% with more complex elements in the HVs. Duan et al. (2022a,b) reach a slightly higher accuracy of 85.47 and 87.11% by mapping the HDC model to an equivalent (B)NN. We can conclude that our proposed HDC method outperforms the native HDC methods but achieves a slightly lower accuracy than the hybrid and multi-bit HDC methods.

In the same way as for the MNIST data set, we compare our obtained result for the Fashion-MNIST data set to lightweight *Non-HDC* methods. Xiao et al. (2017) report accuracies in the range of 51.1–89.7% for several traditional ML methods. The performance of binary SNN ranges from 87.3 to 92.0% (Kheradpisheh et al., 2021; Mirsadeghi et al., 2023). While we are not able to outperform the binary SNNs, our obtained result of 84.62% for Fashion-MNIST is seen to be on par with traditional ML methods.

### 4.3 Robustness analysis

After selecting the hyperparameters yielding the best validation accuracy with a 10-fold CV, the proposed encoding approach is more robust to images corrupted with noise and blur compared to the baseline encoding approach (Supplementary Table S4). Especially for the shot noise and impulse noise corruption, the average test accuracy is fairly equivalent to the average test accuracy achieved on non-corrupted images. For spatter, the average test accuracy is slightly lower but the proposed approach is still able to identify around 81.22% of the test images accurately. The average test accuracy drops the most for the glass blur and motion blur corruption where the proposed approach can classify respectively 57.63 and 39.81% of the images correctly. Still, this is an improvement of 38.42% for glass blur and 28.32% for motion blur compared to the baseline HDC encoding approach. Therefore, we can conclude that the HDC classifier with our proposed encoding approach after hyperparameter selection shows high robustness to noise and blur with an average accuracy of 73.20% across five different corrupted test sets.

### 4.4 Future research

As future work, we envisage evaluating and extending the proposed encoding approach for application to grayscale and color images, investigating the use of hierarchical (multi-layer) patches with HDC encoding and further extensions of the *local linear mapping* concept for position encoding.

Also, it may be analyzed how the HDC framework can be made even more robust to noise and corruption such as glass blur and motion blur.

## 5 Conclusion

We introduce a novel lightweight approach to encode binarized images that preserves the similarity of patterns at nearby locations while relying only on native HD arithmetic vector operations, and not making use of external methods for feature extraction. The approach uses point of interest selection to derive local features of the image and *local linear mapping* to encode the location of these local features in the image. After selecting the best settings for the two introduced hyperparameters with 10-fold cross-validation, an accuracy of 97.92% is reached on the test set for the MNIST data set and 84.62% for the Fashion-MNIST data set. These results outperform other studies using native HDC with different encoding approaches and are on par with more complex hybrid HDC models and lightweight binarized neural networks. The proposed encoding approach also demonstrates higher robustness to noise and blur compared to the baseline encoding.

## Data availability statement

The original contributions presented in the study are included in the article/Supplementary material, further inquiries can be directed to the corresponding author.

## Author contributions

LS: Conceptualization, Methodology, Software, Visualization, Writing – original draft. WV: Conceptualization, Methodology, Writing – review & editing. IT: Writing – review & editing. SL: Funding acquisition, Supervision, Writing – review & editing.

## Footnotes

^1^A list of used symbols can be found in Supplementary Table S1.
