# Supplementary material for: An encoding framework for binarized images using hyperdimensional computing
Source: Front Big Data. 2024 Jun 14;7:1371518. doi: 10.3389/fdata.2024.1371518 (PMC11214273; doi:10.3389/fdata.2024.1371518)
Supplement: Supplementary file 1 [file Data_Sheet_1.pdf]

Supplementary Material

1 HDC CLASSIFIER

The description of the HDC classifier below is taken from our previous work (Smets et al., 2023).

(1) Initial prototype construction. Sample HVs $\mathbf{s}_i$ belonging to the same class l are bundled to form a class bundle $\mathbf{C}_l$ representing the considered class:

$$\mathbf{C}_l = \bigoplus_{i=1}^{m} \{\mathbf{s}_i | y_i = l\} \tag{S1}$$

with y_i the ith sample's class. After binarization of the class bundle $\mathbf{C}_l$, the lth class prototype $\mathbf{c}_l = [\mathbf{C}_l]$ is obtained and stored in the Associative Memory (AM). This is repeated for each class present in the dataset.

(2) Training. The HDC classifier predicts the class for all training samples by calculating the similarity between the training sample's HV $\mathbf{s}_i$ and each class prototype $\mathbf{c}_k$ stored in the AM. The predicted class $\hat{y}_i$ of the input sample is the class with the highest similarity to the input sample's HV:

$$\hat{y}_i = \arg\max_k s(\mathbf{s}_i, \mathbf{c}_k) \tag{S2}$$

If the predicted class is correct (i.e., $\hat{y}_i = y_i = l$), nothing happens. However, if the sample HV $\mathbf{s}_i$ is wrongly classified (i.e., $\hat{y}_i \neq y_i$), it is bundled again in the class bundle of the correct class $\mathbf{C}_l$ and bundled out of the class bundle of the wrong class $\mathbf{C}_{\hat{l}}$ (Imani et al., 2017, 2019a):

$$\mathbf{C}_l = \mathbf{C}_l \oplus \mathbf{s}_i \tag{S3}$$

$$\mathbf{C}_{\hat{l}} = \mathbf{C}_{\hat{l}} \ominus \mathbf{s}_i \tag{S4}$$

with $\ominus$: $\mathcal{B} \times \mathcal{H} \to \mathcal{B}$: $(\mathbf{B}, \mathbf{v}) \to \mathbf{B} - \mathbf{v}$ the bundling out operation which is element-wise subtraction. As such, the class prototypes are adjusted to better classify wrongly classified samples. This updating scheme could be seen as analogous to the multi-class perceptron algorithm (Rosenblatt, 1958; Thomas et al., 2021; Shalev-Shwartz and Shai, 2014).
The training procedure is performed iteratively until either a predefined accuracy on the training set is reached or either a predefined number of iterations is performed. After each iteration, the updated class bundles are binarized with the majority rule into updated class prototypes to be used in the next iteration or finally, in the inference phase.

(3) Inference. The ith test sample is encoded in a query HV $\mathbf{q}_i$ following the same encoding procedure as for the training samples. The predicted class is obtained similarly as during the training procedure with Equation S2 where $\mathbf{s}_i = \mathbf{q}_i$, i.e., the predicted class label of the test sample is the class with the highest similarity to the test samples's HV $\mathbf{q}_i$.

2 SUPPLEMENTARY TABLES AND FIGURES

2.1 List of symbols

A summary of notation can be found in Table S1.

Table S1. List of symbols.

Symbol	Definition	Symbol	Definition
f	feature vector in input space	L	number of levels in ordinal/discrete data
n	number of bundled elements	j	$1...n$
D	HD vector dimension	d	$1...D$
s	similarity	h	Hamming distance
$\mathbf{v}$	vector in HD space $\mathcal{H}$	$\mathbf{B}$	bundle in HD space $\mathcal{B}$
$\mathbf{s}$	sample vector	$\mathbf{S}$	sample bundle
$\mathcal{H}$	vector HD space, $\{0,1\}^D$	$\mathcal{B}$	bundle HD space, $\mathbb{N}^D$
$\oplus$	bundling operator	$[.]$	majority rule
$\otimes$	binding operator	ρ	permutation operator
I	input image	T_{bin}	binarization threshold
I_{bin}	binarized image I	p	flattened image I_{bin}
w	width of image I	h	height of image I
P	patch of I_{bin}	z	patch size
$I_{bin}[x,y]$	value of pixel at position (x,y) in I_{bin}	$p[x]$	value of pixel at position x in p
$P[x,y]$	value of pixel at position (x,y) in patch P	S	number of splits in *local linear mapping*
ρ^i	permutation applied i times		
ρ_X	unique permutation for x-axis in I	ρ_Y	unique permutation for y-axis in I
$\mathbf{v}_{I_{bin}[x,y]}$	HV representing pixel value $I_{bin}[x,y]$	$\mathbf{v}_{p[x]}$	HV representing pixel value $p[x]$
$\mathbf{v}_x$	HV representing position x in p or I	$\mathbf{v}_y$	HV representing position y in I
$\mathbf{x}$	unique random HV for x-axis in I	$\mathbf{y}$	unique random HV for y-axis in I
$\mathbf{v}_{output}$	HV of (last) layer of a neural network	n	number of features
$\mathbf{v}_i$	HV representing the ith feature	$\mathbf{v}_{f[i]}$	HV representing the value of the ith feature
$\mathcal{P}$	set of (x,y) positions of POIs	IM	IM storing binary pixel values
$CIM_{x,z}$	CIM storing position vectors for x-axis in patch P with size z	$CIM_{y,z}$	CIM storing position vectors for y-axis in patch P with size z
$CIM_{x,w}$	CIM storing position vectors for x-axis in image I with width w	$CIM_{y,h}$	CIM storing position vectors for y-axis in image I with height h

Note: HD = hyperdimensional, HV = hypervector, (C)IM = (continuous) item memory.

2.2 10-fold cross-validation results

Table S2 provides the detailed results for the 10-fold cross-validation on the MNIST and Fashion-MNIST data sets for the pixel-wise encoding with varying number of splits S used in *local linear mapping*.

The detailed results for the 10-fold cross-validation on the MNIST and Fashion-MNIST data sets for POI encoding with varying patch size z and number of splits S used in *local linear mapping* are shown in Table S3.

2.3 Robustness analysis

Table S4 provides the detailed results for the robustness analysis on the MNIST-C data sets for the baseline pixel-wise encoding with *linear mapping* (i.e., *local linear mapping* with $S = 1$) and the best

Table S2. Accuracy (%) on the training and validation set and the number of iterations needed to reach the best training accuracy, averaged over the ten folds of 10-fold cross-validation for the MNIST and Fashion-MNIST data sets and for the different settings of the number of splits S used in *local linear mapping*.

	**MNIST**			**Fashion-MNIST**		
S	Training Accuracy	Validation Accuracy	Iteration	Training Accuracy	Validation Accuracy	Iteration
1	61.87 (± 1.04)	60.78 (± 1.57)	590 (± 233)	62.80 (± 1.20)	62.65 (± 1.71)	390 (± 300)
2	77.14 (± 3.35)	75.92 (± 3.45)	754 (± 251)	70.88 (± 0.29)	70.21 (± 0.79)	576 (± 190)
3	86.21 (± 2.08)	84.54 (± 2.30)	555 (± 332)	74.55 (± 0.53)	73.69 (± 0.67)	626 (± 289)
4	93.27 (± 0.80)	91.23 (± 0.95)	545 (± 287)	77.37 (± 0.64)	75.63 (± 0.82)	628 (± 197)
5	93.74 (± 0.67)	91.89 (± 0.99)	219 (± 95)	78.25 (± 0.79)	76.63 (± 0.90)	606 (± 248)
6	94.01 (± 0.61)	92.15 (± 0.60)	195 (± 217)	79.14 (± 0.61)	77.41 (± 0.73)	628 (± 229)
7	94.69 (± 0.98)	92.67 (± 0.66)	121 (± 92)	79.25 (± 1.57)	77.42 (± 1.36)	673 (± 276)
8	95.11 (± 0.83)	92.73 (± 1.16)	220 (± 204)	78.50 (± 1.01)	76.76 (± 0.89)	564 (± 257)
9	95.68 (± 0.77)	**93.21** (± 0.61)	191 (± 102)	79.74 (± 1.35)	77.92 (± 1.35)	380 (± 250)
28	94.56 (± 0.44)	90.53 (± 0.87)	88 (± 52)	84.64 (± 0.70)	**80.98** (± 0.82)	758 (± 230)

Note: Data are *mean* (± *standard deviation*) and in **bold** is the best validation accuracy for each data set.

Table S3. Accuracy (%) on the training and validation set and the number of iterations needed to reach the best training accuracy, averaged over the ten folds of 10-fold cross-validation for the MNIST and Fashion-MNIST data sets and for the different settings of the number of splits S used in *local linear mapping* and of the patch size z used in POI encoding.

		**MNIST**			**Fashion-MNIST**		
S	z	Training Accuracy	Validation Accuracy	Iteration	Training Accuracy	Validation Accuracy	Iteration
1	3	78.93 (± 1.22)	78.41 (± 1.91)	599 (± 308)	66.41 (± 0.43)	65.88 (± 0.62)	576 (± 243)
1	5	87.75 (± 0.68)	87.21 (± 1.87)	673 (± 158)	73.76 (± 0.66)	73.23 (± 0.76)	866 (± 90)
1	7	91.59 (± 2.29)	90.87 (± 2.39)	459 (± 300)	77.93 (± 0.84)	77.29 (± 1.01)	730 (± 252)
2	3	87.84 (± 1.57)	87.13 (± 1.92)	314 (± 314)	77.10 (± 1.84)	76.50 (± 1.66)	657 (± 303)
2	5	97.15 (± 0.30)	96.31 (± 0.44)	106 (± 53)	80.86 (± 1.74)	80.11 (± 1.69)	783 (± 205)
2	7	99.03 (± 0.26)	97.39 (± 0.38)	214 (± 100)	83.48 (± 1.04)	82.39 (± 1.12)	860 (± 115)
3	3	94.21 (± 1.29)	93.66 (± 1.57)	335 (± 391)	78.73 (± 2.05)	78.19 (± 2.11)	678 (± 229)
3	5	97.41 (± 0.79)	96.23 (± 0.86)	421 (± 302)	82.59 (± 0.45)	81.52 (± 0.57)	812 (± 210)
3	7	98.75 (± 0.48)	97.32 (± 0.43)	204 (± 147)	83.36 (± 1.19)	82.14 (± 1.25)	816 (± 238)
4	3	91.38 (± 3.56)	90.99 (± 3.45)	144 (± 301)	82.77 (± 0.65)	81.70 (± 1.00)	729 (± 265)
4	5	99.12 (± 0.11)	97.30 (± 0.23)	262 (± 84)	84.56 (± 0.84)	83.58 (± 0.85)	724 (± 196)
4	7	99.40 (± 0.14)	**97.56** (± 0.29)	188 (± 14)	84.35 (± 0.89)	83.32 (± 1.09)	623 (± 197)
5	3	95.38 (± 2.43)	94.61 (± 2.69)	74 (± 47)	83.46 (± 0.79)	82.36 (± 0.99)	851 (± 112)
5	5	99.14 (± 0.22)	97.38 (± 0.40)	180 (± 35)	84.89 (± 0.73)	83.79 (± 0.70)	723 (± 145)
5	7	99.47 (± 0.25)	97.53 (± 0.35)	176 (± 35)	84.89 (± 0.63)	83.87 (± 0.76)	599 (± 229)
6	3	97.57 (± 0.55)	96.33 (± 0.49)	84 (± 30)	84.20 (± 0.76)	83.11 (± 1.05)	763 (± 223)
6	5	99.41 (± 0.21)	97.21 (± 0.37)	186 (± 39)	85.47 (± 0.79)	84.26 (± 0.73)	685 (± 224)
6	7	99.63 (± 0.30)	97.35 (± 0.34)	168 (± 40)	86.14 (± 0.49)	84.73 (± 0.64)	692 (± 205)
7	3	96.97 (± 1.12)	95.72 (± 0.73)	61 (± 27)	84.66 (± 0.32)	83.27 (± 0.57)	756 (± 214)
7	5	99.43 (± 0.27)	97.19 (± 0.42)	162 (± 39)	85.97 (± 0.45)	84.60 (± 0.65)	686 (± 219)
7	7	99.53 (± 0.36)	97.33 (± 0.33)	150 (± 49)	86.15 (± 0.57)	84.62 (± 0.56)	625 (± 200)
8	3	98.00 (± 0.62)	96.34 (± 0.53)	96 (± 46)	85.68 (± 0.76)	84.21 (± 0.91)	822 (± 193)
8	5	99.25 (± 0.32)	96.97 (± 0.32)	116 (± 43)	86.06 (± 0.87)	84.50 (± 0.71)	734 (± 261)
8	7	99.54 (± 0.35)	97.25 (± 0.34)	146 (± 50)	86.92 (± 0.63)	**85.28** (± 0.71)	661 (± 230)
9	3	98.48 (± 0.49)	96.43 (± 0.50)	138 (± 53)	85.94 (± 0.66)	84.32 (± 0.83)	817 (± 167)
9	5	99.27 (± 0.21)	96.84 (± 0.50)	106 (± 33)	86.49 (± 0.72)	84.96 (± 1.02)	867 (± 113)
9	7	99.39 (± 0.30)	97.05 (± 0.42)	116 (± 42)	86.93 (± 0.54)	85.21 (± 0.69)	763 (± 151)
28	3	97.39 (± 0.75)	93.34 (± 0.66)	50 (± 9)	81.94 (± 1.43)	80.24 (± 1.20)	306 (± 315)
28	5	99.38 (± 0.28)	94.06 (± 0.63)	98 (± 23)	85.25 (± 1.17)	82.34 (± 1.16)	560 (± 289)
28	7	99.59 (± 0.15)	94.40 (± 0.60)	98 (± 4)	86.23 (± 0.64)	83.02 (± 0.63)	634 (± 165)

Note: Data are *mean* (± *standard deviation*) and in **bold** is the best validation accuracy for each data set.

Table S4. Accuracy (%) on the original and five selected corrupted test sets, averaged over ten independent runs for the MNIST-C data set with the baseline hyperparameters ($S = 1$ and no POI selection) and the best hyperparameters ($S = 4$ and $z = 7$). The last row contains the average test accuracy across all corrupted test sets for each setting of hyperparameters.

	**MNIST-C**	
**Corruption**	**Baseline hyperparameters**	**Best hyperparameters**
Identity	62.32 (± 0.63)	97.92 (± 0.07)
Glass Blur	19.21 (± 10.3)	57.63 (± 2.12)
Impulse Noise	57.55 (± 1.06)	90.67 (± 0.66)
Motion Blur	11.49 (± 0.82)	39.81 (± 1.91)
Shot Noise	42.21 (± 8.13)	96.68 (± 0.13)
Spatter	36.72 (± 5.08)	81.22 (± 0.22)
Average	33.44 (± 18.39)	73.20 (± 23.86)

Note: Data are *mean* (± *standard deviation*).

hyperparameters for POI encoding (i.e., *local linear mapping* with $S = 4$ and window size around POI $z = 7$).

2.4 Comparison to the State-of-the-Art

Table S5 compares our obtained results to the results of studies found in the literature for the MNIST and Fashion-MNIST data sets.

Table S5. Comparison of our obtained results to the results from studies found in the literature for the MNIST and Fashion-MNIST data sets.

Category	Method	Reference	Accuracy	
			MNIST	Fashion-MNIST
Native HDC	Permutation 1D	Manabat et al. (2019)	79.87	-
		Hassan et al. (2022)	86	-
	Binding 1D	Kazemi et al. (2021)	85	-
		Chang et al. (2021)	87	-
		Duan et al. (2022b)	87.38	79.24
		Bosch et al. (2022)	88.3	-
		Hsieh et al. (2021)	88.8	-
		Chuang et al. (2020)	88.92	-
		Duan et al. (2022a)	89.28	80.26
		Ma and Jiao (2022)	90.93	-
		Hernández-Cano et al. (2021)	91	-
		Zou et al. (2021b)	92	-
	Permutation & Binding 1D	Khaleghi et al. (2022)	94.0	-
	***Local linear mapping* and POI**	**Ours**	**97.92**	**84.62**
Adaptive HDC	Binding 1D	Hernández-Cano et al. (2021)	97.5	-
Hybrid HDC	Elementary Cellular Automata	Karvonen et al. (2019)	74.06	-
	Spiking Neural Network	Zou et al. (2021a)	90.5	-
	Binary Neural Network	Duan et al. (2022a)	94.74	87.11
		Yan et al. (2023)	97.25	-
	Random Fourier Features	Yu et al. (2022)	95.4	84.0
	Neural Network	Duan et al. (2022b)	92.72	85.47
		Liang et al. (2022)	94.8	-
		Ma and Jiao (2022)	96.71	-
		Poduval et al. (2021)	99	-
		Kussul and Baidyk (2004)	99.2	-
		Kussul et al. (2006)	99.5	-
	Manifold learning	Zou et al. (2021b)	97.5	-
	Local Binary Pattern	Rachkovskij (2022)	98.5	-
Multi-bit HDC		Kazemi et al. (2021)	95.5	-
		Yu et al. (2022)	96.6	87.4
		Imani et al. (2019b)	98	-
		Chuang et al. (2020)	98.09	-
		Kim et al. (2021)	98.2	-
Non-HDC	Gaussian Naive Bayes	Xiao et al. (2017)	52.4	51.1
	Extra-Trees	Xiao et al. (2017)	84.7	77.5
	Passive Aggressive	Xiao et al. (2017)	88.0	77.6
	Decision Tree	Xiao et al. (2017)	88.6	79.8
	Linear Perceptron	Xiao et al. (2017)	88.7	78.2
	Linear Classifier with SGD[1] training	Xiao et al. (2017)	91.4	81.9
	Logistic Regression	Xiao et al. (2017)	91.7	84.2
	Linear Support Vector Classification	Xiao et al. (2017)	91.9	83.6
	K-Nearest Neighbors	Xiao et al. (2017)	95.9	85.4
	Gradient Boosting	Xiao et al. (2017)	96.9	88.0
	Random Forest	Xiao et al. (2017)	97.1	87.3
	Multi-Layer Perceptron	Xiao et al. (2017)	97.2	87.1
	HOG[2] Features with AdaBoost	Kim et al. (2017)	97.5	-
	Support Vector Classification	Xiao et al. (2017)	97.8	89.7
	Binary Neural Network	Narodytska et al. (2018)	95.7	-
		Chi and Jiang (2018)	97	-
		Yang et al. (2017)	97	-
		Yan et al. (2023)	97.71	-
		McDanel et al. (2017)	97.86	-
		Cheng et al. (2015)	97.88	-
		Ghasemzadeh et al. (2018)	98.29	-
		Jokic et al. (2018)	98.4	-
		Umuroglu et al. (2017)	98.4	-
		Valavi et al. (2018)	98.60	-
		Kim and Smaragdis (2015)	98.64	-
		Simons and Lee (2019)	98.77	-
		Sun et al. (2018)	98.77	-
		Courbariaux et al. (2015)	98.99	-
		Courbariaux et al. (2016)	99.04	-
	Binary Spiking Neural Network	Kheradpisheh et al. (2021)	97.0	87.3
		Mirsadeghi et al. (2023)	98.6	92.0

Note: In **bold** is the obtained accuracy with the proposed encoding approach for each data set.

[1] SGD = Stochastic Gradient Descent

[2] HOG = Histogram of Oriented Gradients
